# Plasmid DNA nicking- a Novel Activity of Soybean Trypsin Inhibitor and Bovine Aprotinin

**DOI:** 10.1038/s41598-019-48068-6

**Published:** 2019-08-12

**Authors:** M. Rafiq Islam, Kelvin Ihenacho, Jae Whan Park, I. Sakif Islam

**Affiliations:** Laboratory of Biochemistry, Northwest Missouri State University, 7314 N. Tullis Ave, Kansas City, Missouri 64158 United States of America

**Keywords:** DNA, DNA restriction-modification enzymes, Single-strand DNA breaks

## Abstract

Protease inhibitors, such as trypsin inhibitor, serum alpha-1 antitrypsin, or liver aprotinin, are a class of proteins that competitively bind and block the catalytic activity of proteolytic enzymes with wide ranging biological functions. A significant number of protease inhibitors have also been shown to possess antimicrobial activity, presumed to contribute in defense against pathogenic microorganisms as plants with higher levels of protease inhibitors tend to exhibit increased resistance towards pathogens. Two proposed mechanisms for the antimicrobial activity are combating microbial proteases that play roles in disease development and disruption of microbial cell wall & membrane necessary for survival. Here we show for the first time a novel activity of soybean trypsin inhibitor and bovine aprotinin that they nick supercoiled, circular plasmid DNA. A number of experiments conducted to demonstrate the observed DNA nicking activity is inherent, rather than a co-purified, contaminating nuclease. The nicking of the plasmid results in markedly reduced efficiencies in transformation of *E. coli* and transfection of HEK293T cells. Thus, this work reveals yet a new mechanism for the antimicrobial activity by protease inhibitors.

## Introduction

Widely distributed among plants, animals and microorganisms^1^, protease inhibitors (PIs), particularly trypsin inhibitors (TIs), are found in many body fluids including serum, pancreatic enzyme secretion, eggs and seeds. Reported functions include regulation and control of endogenous proteinases and enzymes in serum^2^, as sporamin-like storage protein or nutrient for the developing embryo in eggs^3^, and protection against consumption by animal predators (by affecting protein digestion) in seeds (antinutritive effect)^4^ or protection from microbial attack in many^5,6^. Soybean trypsin inhibitor (STI) showed anticarcinogenic activity by down-regulating c-myc expression^7,8^. Serum α1 anti-trypsin reported to play immunoregulatory function, inhibit neutrophil superoxide production, induce IL-1 receptor antagonist expression, and reduce infection by Cytosporidium parvum in rats and Pseudomonas aeruginosa in cystic fibrosis patients^9,10^. Trypsin inhibitors from the seeds of Chinese white cabbage and bottle gourd possess antimicrobial (AM) activities^11^. Double headed inhibitors from broad beans and potato tubers showed antifungal activity^5,11^. Inhibitors Mungoin from mung bean and Potide G from potato tubers exhibited both AM and antifungal activities^5,12^. A serine protease inhibitor found in liver, pancreas and blood, Aprotinin (also known as Antilysin(e), Basic pancreatic trypsin inhibitor (BPTI); Kallikrein-trypsin inactivator) also exhibits AM activity against a variety of microorganisms including influenza virus and fungus^13,14^. Its PI activity against plasmin is exploited to slow down fibrinolysis, the process to breakdown blood clots, in order to reduce the need of blood transfusions during complex surgery^15^. Similar to STI, which is a monomeric protein consisting of 181 amino acids with two disulfide bridges (20.1 kDa)^16^, aprotinin is also a monomeric protein composed of 58 amino acids (6.5 kDa) with three crosslinking disulfide bridges^17^.

Pathogens are known to produce extracellular proteinases which are considered to play roles in disease development. Thus AM activity of proteinase inhibitors (PI) may be simply a consequence of combating these proteinases in pathogens^1,18^. In fact, PIs do accumulate in plants in response to insect lesion^19^ or pathogenic microbes^20^. Likewise, concentration of AM lactoferrin rises sharply during infection of mammary gland^21^. On the other hand, a wide variety of PIs do not possess any AM activity^22^. In addition, accumulation in response to mechanical wounding^23^ or UV-radiation^24^, arguing against countering pathogenic proteases as the sole mechanism for AM activity. A recent report on structure-function relationship of ORB, a short inhibitor with AM activity isolated from skin secretions of Odorrana grahami, proposed AM activity was independent of TI activity^25^. Similar observation was made from structural studies with bovine aprotinin^26,27^. AM proteins were also thought to act through cell wall and/or membrane disruption but recent experiments have revealed a diversity of mechanisms^15^. Here, we reveal for the first time a novel activity of soybean trypsin inhibitor and bovine aprotinin as nicking plasmid DNA and reducing its efficiency. This may provide yet a new mechanism for AM activity by PIs.

## Results

### Plasmid DNA nicking by Soybean Trypsin Inhibitor (STI)

**Figure 1:**
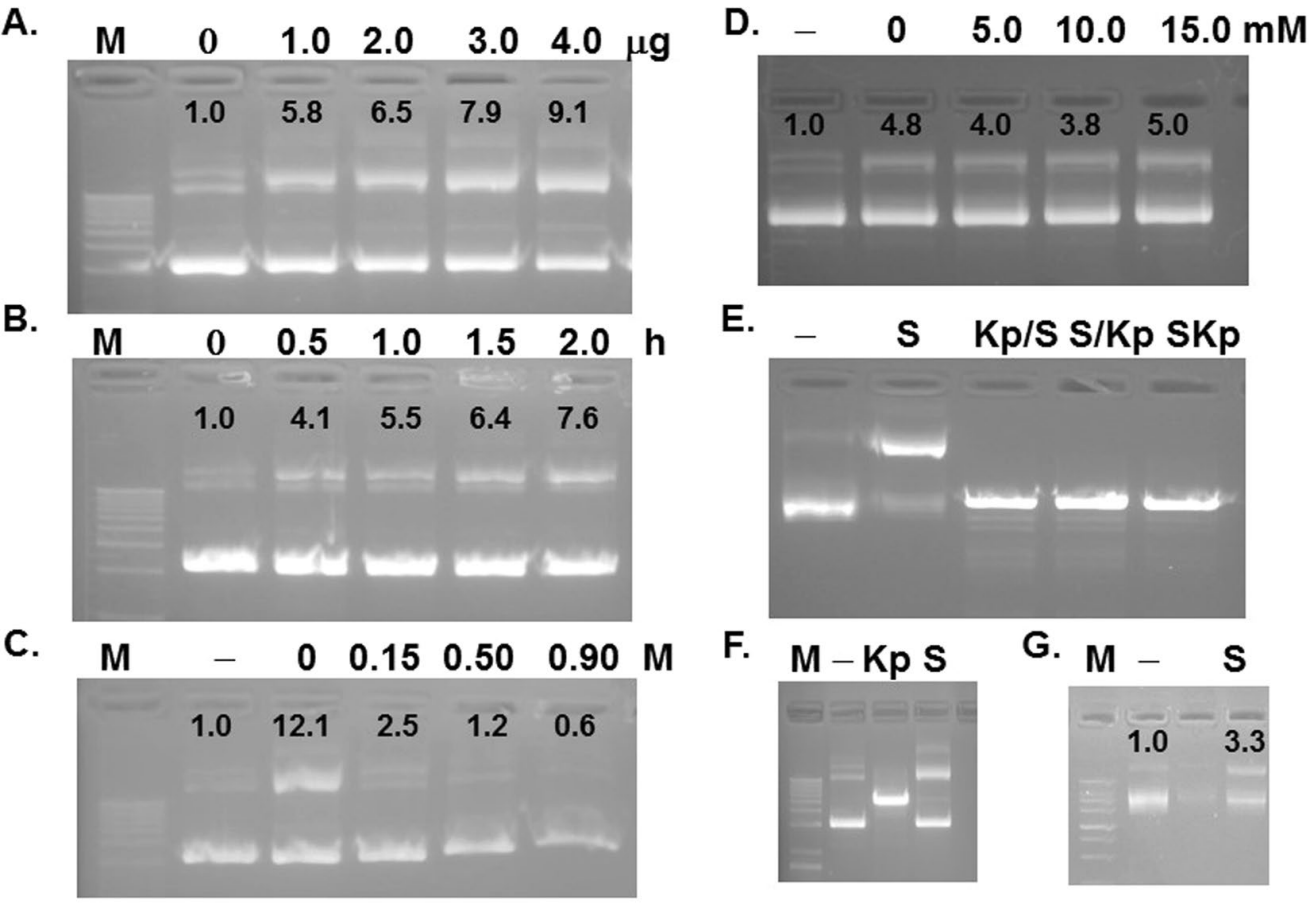
Soybean Trypsin Inhibitor (STI) affects migration pattern of plasmid pGL3. pGL3 plasmid (0.5 ug) in Buffer A (25 mM Tris-acetate (pH 7.5), 100 mM potassium acetate, 10 mM magnesium acetate) was incubated with 0–4 ug of STI (**A**), for 0–2 h (**B**), in the presence of 0-0.9 M NaCl (**C**) or 0-15 mM EDTA (**D**). (**E**) prior to incubation with STI (S), the plasmid was linearized with KpnI (Kp/S), or vice-versa (S/Kp) or co-incubated with both (SKp). (**F**) longer run of STI or KpnI treated plasmid. (**G**) STI treated or untreated pGL3 was purified using a PCR reaction purification kit or phenol-chloroform extraction prior to run on a gel. All samples were analyzed on 2% agarose gels stained with ethidium bromide. The intensity of the slower moving bands was quantified using NIH ImageJ. The intensity of the control (− or 0 lane) was set to 1.0. The intensity of the slower migrating band increases with STI amount and incubation time, but decreases in the presence of NaCl, while presence of EDTA has no effect. The STI induced slower migrating band disappears with linearization using digestion with Kpn I, however, remains intact following purification by phenol-chloroform extraction or other method. Samples in each gel were prepared in parallel. All gels were processed using Microsoft Word “Corrections” for brightness and contrast applied equally across the entire image including controls. The full length gels are presented as Supplementary Information with corresponding figure numbers as in the main article.

The plasmid used in this study was pGL3 basic vector (4818 bp) containing ampicillin resistance for selection with a multiple cloning site upstream of the luciferase (Luc) reporter and downstream of the origin of replication (ori) lacking a true promoter. After incubation of this plasmid DNA with STI at 37 °C followed by electrophoresis on a 2% agarose gel, we noticed a slower migrating band starting to appear. The intensity of this slower migrating band increased from 5.8 to 9.1 fold with incubation with increasing STI amount (Fig. 1A); the intensity of the faster migrating band, on the other hand, decreased proportionately. Enrichment of the slower migrating band also depended on the incubation time (Fig. 1B)- 4.1 fold in 30 min to 7.6 fold in 2 h. Increasing NaCl concentration in the reaction mixture, however reduced its accumulation down to 0.6 fold in 0.9 M NaCl (Fig. 1C) - suggesting an interaction between STI and plasmid DNA was required for appearance of the slower migrating band and the interaction was disrupted at higher ionic strength. In fact, the interaction did not require any metal ions as presence of EDTA in the reaction mixture had no effect on the intensity of slow migrating band (Fig. 1D).

Using the sole Kpn I site, when we linearized the plasmid DNA prior to (KpS), after (SKp) or simultaneously (S/Kp) with STI treatment, all the treated DNAs gave a single band with similar migration pattern, with no slower moving band (Fig. 1D), suggesting the STI effect cannot be detected in linear DNA migration. Several points are apparent from this result: the slower migrating band produced by STI treatment collapsed into a linearized band, which was running intermediate between the faster and slower migrating bands (Fig. 1F), although in a longer run, the linearized DNA eventually migrated past the faster moving band as reported previously^28^. This study on electrophoretic migration pattern of plasmid DNA concluded the fastest moving band as closed circular, supercoiled DNA, the slower migrating band as nicked DNA (one strand break), and the intermediate migrating band as linearized (both strand break) DNA^28,29^. Thus, the apparently nicked DNA band produced by STI treatment (Fig. 1E) collapsed upon linearization as the nick in linearized DNA cannot be separated in a usual agarose gel without strand separation.

As DNA complex with protein may migrate slowly^30^, to rule out the possibility that the slower migrating band was a STI-DNA complex, the STI treated plasmid was purified using phenol-chloroform extraction or PCR reaction cleanup kit to remove proteins. The purified DNA from the STI treated plasmids still retained the slower migrating band (Fig. 1G), indicating it was not due to a complex formation with STI, rather a permanent change was introduced by STI in the plasmid DNA. In another experiment, following STI treatment one-half of the reaction mixture was boiled, then digested with proteinase K to remove STI. Upon gel electrophoresis, intensity of the slower moving band remained similar compared to the other half (data not shown) further confirming that the slower moving band was not due to STI-DNA complex.

### Nicking activity is sensitive to proteinase K treatment on STI

**Figure 2:**
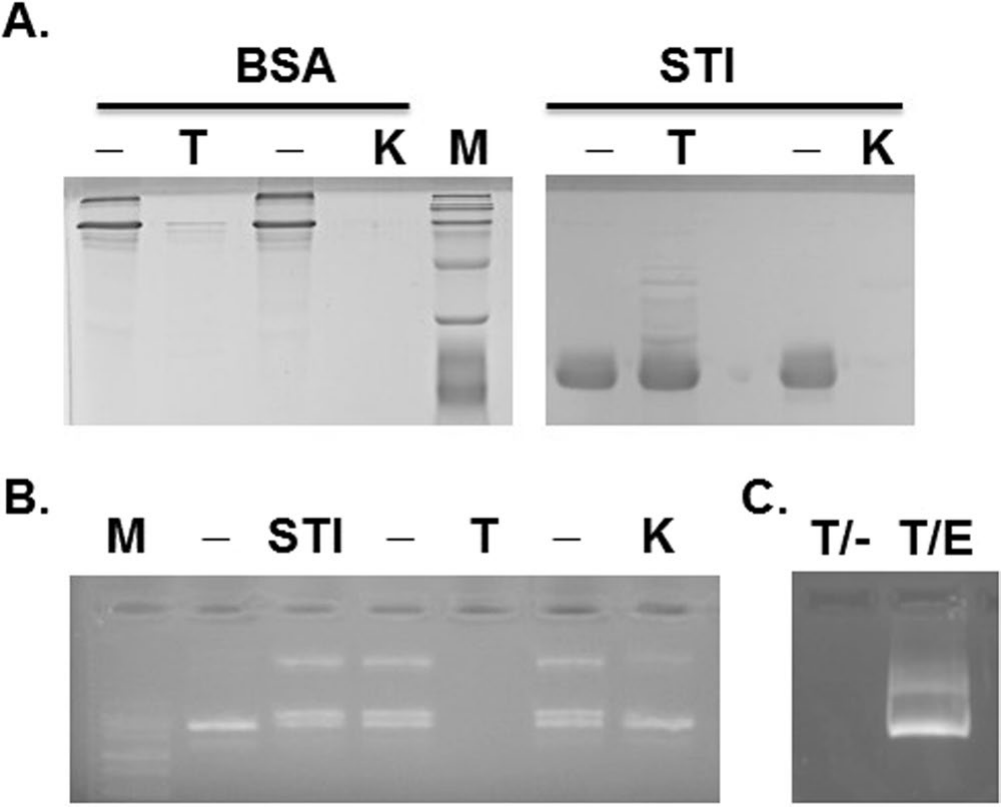
Nicking by STI is persistent with trypsin treatment, but sensitive to proteinase K treatment. (**A**) BSA (as control of digestion) or STI was treated without (−), with trypsin (T) or with proteinase K (K) and analyzed by SDS-PAGE. The enzyme treated or untreated STIs were assayed in DNA nicking activity (**B**). No DNA bands in trypsin treated STI (B, lane T) is due contaminated DNase in trypsin preparation. The DNase in trypsin can be inactivated with incubation with 15 mM EDTA (**C**, lane T/E). Samples in each gel were prepared in parallel. All gels were processed using Microsoft Word “Corrections” for brightness and contrast applied equally across the entire image including controls. The full length gels are presented as Supplementary Information with corresponding figure numbers as in the main article.

STI obtained from Sigma-Aldrich was prepared by ammonium sulfate fractionations along with a series of clarification steps followed by chromatographic purification and indicated a purity of ≥98%^31^. On SDS-PAGE, it appeared as a single band with no other protein band (Fig. 2A, lane STI/−). Nonetheless, it is possible that one or more contaminant proteins is present in the STI preparation, but not enough to be detected on gel or it may be migrating along-side with STI, i.e., of similar size. To eliminate these possibilities, we subjected STI to Trypsin and Proteinase K digestion. As expected, Trypsin did not digest STI, but completely digested BSA used as a control since no corresponding band was visible in the SDS-PAGE gel (Fig. 2A, lane BSA/T). Proteinase K, on the other hand, digested both BSA and STI nearly completely (lanes BSA/K and STI/K). The DNA nicking activity appeared significantly reduced when assayed with Proteinase K treated STI (Fig. 2B, lane K) compared to untreated STI (lane -). Unfortunately, all plasmid DNA incubated with the trypsin treated STI were digested most likely by one or more contaminant nucleases present in Trypsin preparation (Fig. 2B, lane T and Fig. 2C, lane T/−). In fact, this was the case as DNA treated with the Trypsin preparation alone was also completely degraded (not shown).

Since, nucleases and DNA modifying enzymes are usually metal dependent (prefer a divalent ion such as Mg^+2^ or Zn^+2^), and previously we determined that metal chelator EDTA had no effect on STI’s DNA nicking activity (Fig. 1D), we exploited these findings. We incubated trypsin treated STI reaction mixture with EDTA prior to use in plasmid DNA nicking assay. Most DNAs are protected from nuclease digestion and appearance of the slower migrating band was evident (Fig. 2C, lane T/E). Since trypsin might digest contaminant nucleases or DNA modifying enzyme and EDTA might inactivate these enzymes, but neither will affect STI, prior treatment with both thus eliminated the possibility that these enzymes, if present in STI preparation, were responsible for the nicking activity.

Taken together, these results indicated that STI itself is responsible for the observed plasmid nicking.

### Effects of heat, reducing agents, and low pH treatments on STI’s DNA nicking activity

**Figure 3:**
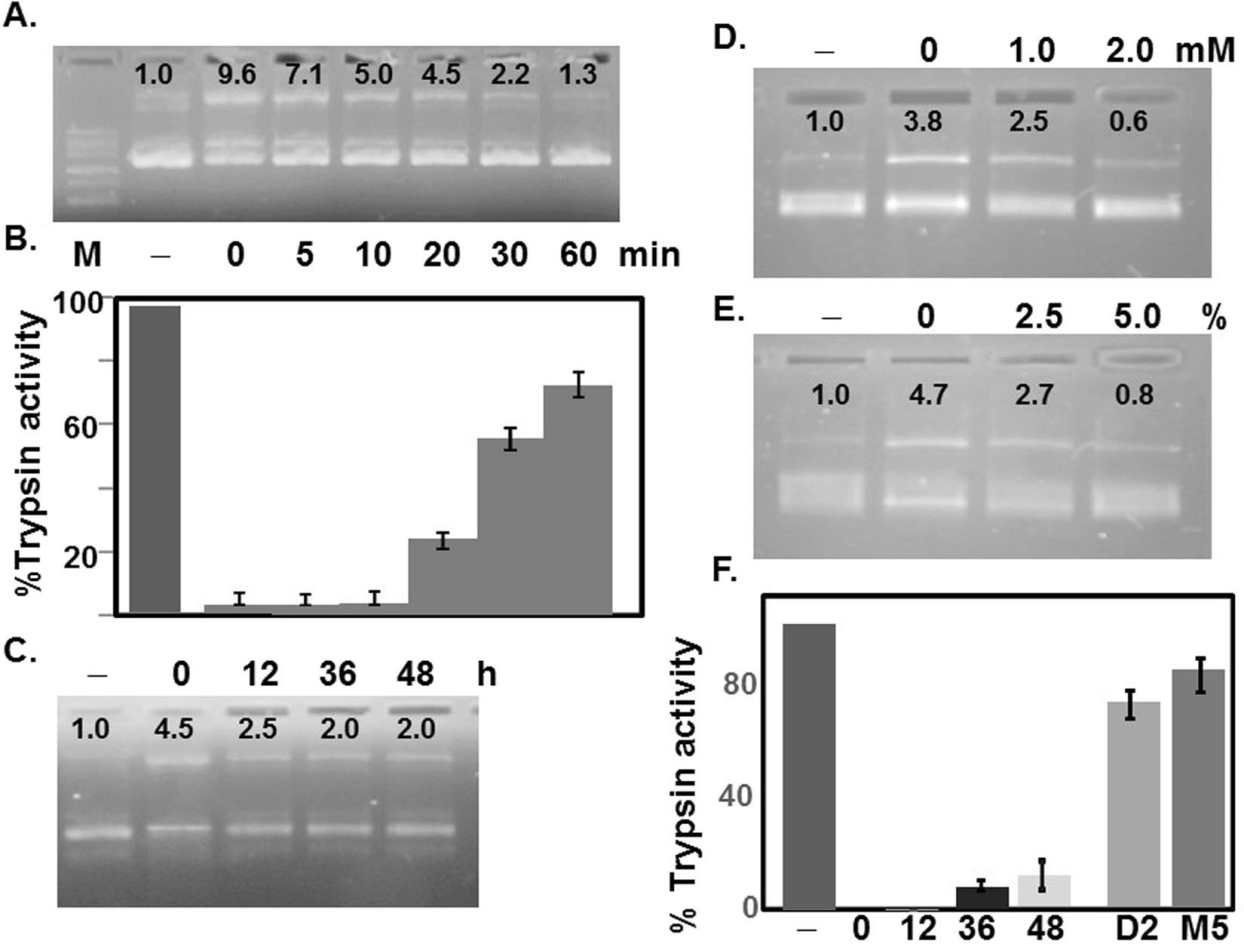
Effects of heat, reducing agents, and low pH treatments on STI’s DNA nicking activity. STI was heated at 90 °C for 0-60 min prior to measuring its DNA nicking (**A**) and trypsin inhibitory activities (**B**). Each bar is the mean ± S.D (**p < 0.005) of a representative experiment performed in triplicate. STI subjected to pH 2 treatment (**C**) for 0-48 h, or reduced with 0-2 mM DTT (**D**) or 0-5% 2-mercaptoethanol (**E**) prior to plasmid nicking (**C**–**E**) and trypsin inhibitory activities were assayed (**F**). Each bar is the mean ± S.D (**p < 0.005) of a representative experiment performed in triplicate. The intensity of the slower moving bands was quantified using NIH ImageJ. The intensity of the control (− or 0 lane) was set to 1.0. Loss of DNA nicking activity is correlated with loss of trypsin inhibitory activity in heat or reducing agents treated, but not in low pH treated STI. Samples in each gel were prepared in parallel. All gels were processed using Microsoft Word “Corrections” for brightness and contrast applied equally across the entire image including controls. The full length gels are presented as Supplementary Information with corresponding figure numbers as in the main article.

Further evidence supporting STI itself possessing plasmid nicking activity was obtained from exploiting a number of unique characteristics of STI. It was shown previously that STI can retain significant trypsin inhibitory (TI) activity after a short period of heat treatment^31,32^. Assuming heat labile, the contaminant protein(s) might denature quickly with heat treatment, leaving TI activity intact. Heated STI samples demonstrated a gradual loss in TI activity with heating period as expected, but retained 50% after 30 min and 15% after 60 min of heating (Fig. 3B). The plasmid DNA treated with these heat-treated STI samples showed a gradual loss in DNA nicking activity (Fig. 3A), which correlated well with the loss of TI activity by heat (Fig. 3B), implicating that the nicking activity is most likely due to STI itself, unless the contaminant(s) is also heat stable. However, most DNA modifying enzymes known are usually heat labile suggested otherwise.

S-S bridge(s) between cysteine residues seem to be important for both TI and AM activities^25^, therefore nicking activity by STI performed in the presence of reducing agent DTT or 2ME revealed markedly reduced nicking activity (Fig. 3D,E). As expected, a similar reduction in the TI activity was also noted (Fig. 3F, D2 & M5) further supporting STI’s involvement in the nicking activity. It also predicts that both (TI and nick) active sites may be located in the same structural domain of STI.

When pre-incubated at pH 2.0 for 48 h, Ovorubin, a trypsin inhibitor without AM activity isolated from apple snail eggs, lost its TI activity completely^4^. In contrast, a similar treatment of STI recorded a little loss in its TI activity (Fig. 3F, 12, 36, 48). However, a significant loss in nicking activity was observed, which mostly occurred within the first 12 h of incubation at pH 2.0 (Fig. 3C). This result suggests either the TI and nicking activities are carried out by different proteins, thus supporting the presence of a contaminant in STI preparation, or STI itself possess both activities but at two different sites in its structure.

### STI purified using FPLC retains DNA nicking activity

**Figure 4:**
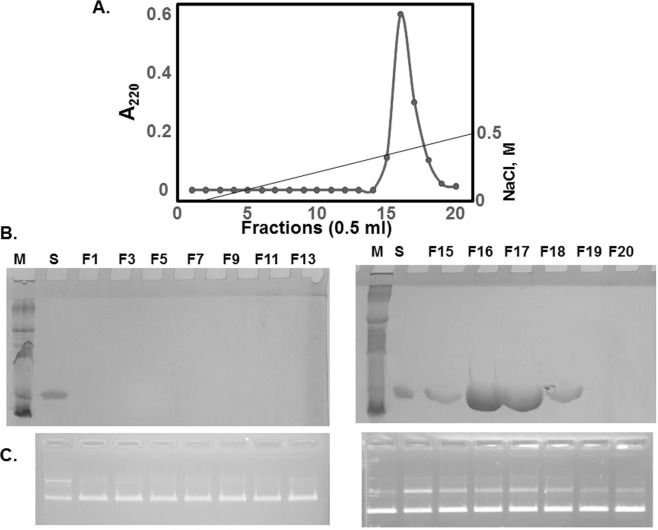
STI purified using FPLC retains DNA nicking activity. (**A**) STI was subjected to purification using FPLC on a Mono Q column in 50 mM Tris-HCl, pH 8.0 buffer and eluted with a linear gradient of 0.5 M NaCl in the same buffer. Four fractions (F15-F18) collected under the only peak as well as every other fractions with no peak were analyzed by SDS-PAGE (**B**) and for DNA nicking activity (**C**) along without (−) or with STI (S). Samples in each gel were prepared in parallel. All gels were processed using Microsoft Word “Corrections” for brightness and contrast applied equally across the entire image including controls. The full length gels are presented as Supplementary Information with corresponding figure numbers as in the main article.

Finally, in fast protein liquid chromatography (FPLC) with detection at 220 nm (peptide bond), STI bound to Mono Q column was eluted as a sole (Fig. 4A) peak, consistent with a single band observed in SDS-PAGE (Fig. 2, lane STI/−), confirming manufacturer’s claim of its high purity. However, co-elution of contaminant protein(s) with STI could not be ruled out. However, SDS-PAGE analysis of the four fractions (F15–F18) collected under the peak each showed a single band (Fig. 4B, lanes F15–F18) compatible with the shape and sharpness of the peak. All four fractions showed DNA nicking activity (Fig. 4C). Analysis of the fractions collected with no peak observed in the FPLC chromatogram showed no protein band on SDS-PAGE, nor plasmid nicking (Fig. 4B,C, lane F1–F13) activity was observed. These results further supported that STI itself, rather than any contaminants may present in it, is responsible for the observed DNA nicking activity.

### DNA nicking activity is inherent to STI

**Figure 5:**
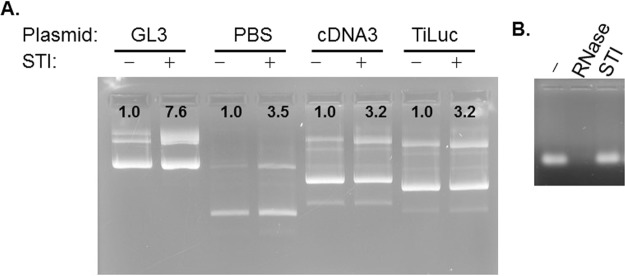
STI nicks other plasmids, and does not possess RNase activity. (**A**) Nicking activity was done as in Fig. 1 with plasmids: pBluescript (PBS), pCDNA3, TiLuc. (**B**) 1 ug of total RNA from HepG2 cells was treated with 1 ug of RNase or 3 ug STI for 1 h at 37 °C, then analyzed on agarose gel stained with EtBr. Samples in each gel were prepared in parallel. The intensity of the slower moving bands was quantified using NIH ImageJ. The intensity of the control (− or 0 lane) was set to 1.0. All gels were processed using Microsoft Word “Corrections” for brightness and contrast applied equally across the entire image including controls. The full length gels are presented as Supplementary Information with corresponding figure numbers as in the main article.

This nicking activity by STI was also observed with other cloning or expression plasmids investigated (Fig. 5A), although the extent of nicking was variable (3.5 fold to 7.5 fold) for each. Since a ribonuclease was isolated with AM activity^33^, we treated RNA isolated from HEK293T cells with STI, however it did not display any RNase activity (Fig. 5B) when examined.

Taken all the results together, it appears that the plasmid nicking activity exhibited by STI is emanated from and inherent to STI rather than contributed by one or more nucleases that might be present as contaminant in the STI preparation.

**Figure 6:**
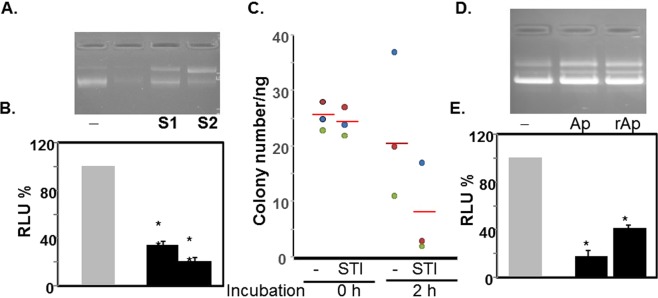
STI treatment lowers transfection and transformation efficiency of plasmid DNA. Following incubation without (−) or with STI (S1 or S2) for 0 or 2 h, the plasmid pGL3-PKD1 (pGL3 inserted with a 200 bp PKD1 proximal promoter to drive expression of Luciferase) DNAs were purified by a PCR reaction purification kit and analyzed by agarose gel (**A**). The purified DNA (0.1 ug) was used in co-transfection with β-galactosidase (0.1 ug) cDNA in HEK293T cells. The expressed luciferase activity in each was assayed and normalized with co-expressed β-galactosidase activity. The normalized RLU value obtained in cells transfected with untreated pGL3-PKD1 was set at 100% (**B**). Each bar is the mean ± S.D (**p < 0.005) of a representative experiment performed in triplicate. (**C**) 50 ng the purified STI-treated DNA was used for transformation of XL1-blue competent cells. Each symbol with matching color in both 0 h and 2 h represents an independent experiment. The bar is the mean of the data in each^41^ (Kueh *et al*. Science 341:670-673, 2013). (**D**) DNA nicking activity by bovine Aprotinin (Ap) and recombinant Aprotinin (rAp) overexpressed in Nicotania. (**E**) Transfection efficiency of the pGL3-PKD1 treated with Ap or rAp. The expressed luciferase activity was assayed and normalized as in B (**p < 0.005). Samples in each gel were prepared in parallel. All gels were processed using Microsoft Word “Corrections” for brightness and contrast applied equally across the entire image including controls. The full length gels are presented as Supplementary Information with corresponding figure numbers as in the main article.

To determine whether another inhibitor with AM activity, but of animal origin, could exhibit plasmid nicking, we used two different preparations of aprotinin: one isolated from bovine pancreas using several chromatographic purification steps^34^ and another a recombinant version purified from overexpressed plant (Nicotania). Both preparations displayed plasmid nicking activity (Fig. 6D, lanes Ap & rAp) similar to STI, however a minor band migrating intermediate between the supercoiled and nicked DNA appeared in both preparations. We speculate that this band was generated by double-strand break (i.e., linearized) at the same or different location. Further studies are needed to confirm the band was whether produced by aprotinin or by contaminant nucleases.

### STI treatment lowers transfection and transformation efficiency of plasmid DNA

To investigate what effect nicking might have on the plasmid, we purified both treated and untreated plasmid using reaction-clean-up kit and then used in functional assays in prokaryotic and eukaryotic cells. It is known that linearization or even nicking reduces transfection and transformation ability^35,36^. Since pGL3 is a promoter-less plasmid, we inserted a 200 bp proximal promoter of PKD1 (Polycystic Kidney Disease 1) gene into MCS just upstream of luciferase reporter to drive luciferase expression^37^. This plasmid was also nicked by STI (Fig. 6A) and we noticed over 60% reduction in transfection efficiency in HEK293T cells comparing expressed luciferase over co-expressed beta-galactosidase in cells transfected with STI or aprotinin treated versus untreated plasmids (Fig. 6B,E). Consistent with this, transformation of *E. coli* XL-1 blue cells by STI treated plasmid was markedly reduced (Fig. 6C). These data demonstrated that treatment with PIs significantly damaged the plasmid and reduced its effectiveness, most likely by reducing replicon number.

### Antimicrobial activity of STI

A wide variety of protease inhibitors obtained from both plant and animal sources show varying degrees of antimicrobial activity against many species. Despite extensive search, we were unable to obtain literature on AM activity of STI, although its anticarcinogenic and other activities were reported^7,8^. Thus, we determined its minimum inhibitory concentration (MIC) and compared with a number of PIs. As presented in Table 1, its activity is comparable to other PIs, but much weaker than aprotinin. However, STI showed a stronger activity (3-fold) against the same bacterial cells harboring a plasmid with ampicillin resistance gene (*Amp*^*r*^), compared to without plasmid.

**Table 1:** Antimicrobial activity of soybean trypsin inhibitor against *E. coli*.

Protein/Peptide	Molecular weight, kDa	MIC (μg/ml (μM)	Comments
Lactoferrin^21^	76.0	1000 (13)	
Lactoferrin tryptic peptide^21^	3.1	12.5 (4)	S. aureus (+): 50 μg/ml
ORB^25^	2.3	3.2 (1.4)	S. aureus (+): 5.8 μg/ml
PT-1^5^	5.6	500 (100)	
AMTI-II^42^	21.2	62.5 (2.5)	
Aprotinin^15,43^	6.5	19.5 (3.0)	
STI	20.1	600 (29.9)	with *Amp*^*r*^ plasmid: 200 μg/ml

## Discussion

In this study, we revealed a novel activity of STI, a Kunitz type inhibitor from soybean selective for Trypsin and Chymotrypsin, that it possesses plasmid DNA nicking activity. Considerable number of experiments exploiting unique characteristics of STI, such as its heat stability, resistance to trypsin or EDTA, and sensitivity to reducing agents and proteinase K digestion were conducted aiming at eliminating the possibility of one or more contaminants that might be present in STI preparation responsible for this activity (Figs 2 and 3). Furthermore, STI purified using FPLC cation exchange column retained the nicking activity (Fig. 4). Slower moving DNA band was not due to a complex formation with STI rather a permanent alteration was introduced (Fig. 1F), and diminished with increased salt concentration (Fig. 1C). Based on these results, we concluded that STI itself possesses the plasmid DNA nicking activity.

Although we cannot rule out presence of a contaminant that might be responsible for the DNA nicking activity of aprotinin, but similar results with two different preparations obtained employing different methods and sources (animal and plant) tend to suggest aprotinin also possesses such activity (Fig. 6D). The treatment with PIs significantly damaged the plasmid and reduced its effectiveness as determined by transformation into bacterial cells and transfection into mammalian cells (Fig. 6B,C,E).

Since aprotinin is one of the earliest known antimicrobial protein, strongly AM potent (MIC 3 μm), relatively smaller in size (58 amino acids mature peptide), and shows activity against bacteria and virus, several studies had been performed to define its AM active site. Following clostripain digestion, one antiviral and three antibacterial peptides were isolated by Pellegrini *et al*.^26,27^. All three peptides, P1–15 (includes PI site Lys15), P18–P39 and P40–58, showed AM activity with peptide P18–39 being the most active. However, each peptide was weaker than the full length aprotinin. The P18–39 segment of the peptide forms an antiparallel β−sheet with short hairpin. AM activity resides on the N-terminal P18–26 (IIRYFYNAK) forming the 1^st^ stand, while the peptide P27–39 forming the 2^nd^ strand had no activity^38^. These show that the AM site in aprotinin is near to or overlap with the PI site but is independent.

Similar study on ORB demonstrated its TI activity, which depended on a core sequence of nine residues with cross-linked Cys at both termini creating a loop (TI loop), can be differentiated from its AM activity, which needed additional residues outside the TI loop^23^. It was proposed that both activities originate from a common ancestor with TI function being the primitive one and the AM function acquired later to control microbial infections. We found treatment of STI with reducing agents markedly reduced its both TI and nicking activities (Fig. 3D–F) indicating S-S cross-link most likely the one across the TI domain between Cys65 and Cys109 is essential for both activities. On the other hand, incubation of STI at pH 2.0 while significantly impaired its nicking activity (Fig. 3C), the TI activity remained intact (Fig. 3F), suggesting the two sites are separate as determined in aprotinin and ORB. TI domain of STI comprises the residues within Cys65-Cys109 with Lys78 being in the active center^39^. Like AM active site in aprotinin and ORB, it is reasonable to assume that the nicking site in STI may reside within this 31 amino acids between Cys65-Cys109. Sequence comparison using Blastp of aprotinin P18-26 peptide (IIRYFYNAK) with two short peptides from ORB^25^ and lactoferrin^21^ having AM activity as well as TI domain of STI was unable to identify any homologous sequence. Thus additional studies will be required to identify sequence in STI responsible for the nicking activity.

Antimicrobial activity of STI measured against Gram-negative *E. coli* cells is comparable to other known protease inhibitors (Table 1). What is interesting is that it was three times more active against the same strain containing a plasmid for ampicillin resistance (Table 1, comments). The latter cells were grown in media containing ampicillin to maintain the plasmid inside cells. Ampicillin inactivates penicillin-binding protein, a glycopeptide transpeptidase, in the periplasm between the outer and inner membranes in Gram-negative bacteria through covalent acylation of its active site serine. Thus, unable to cross-link two strand of peptidoglycan leads to cell wall disruption and ultimate bacterial cell death. Amp resistance (*Amp*^*r*^) plasmid expresses β-lactamase enzyme which confers amp-resistance to cells harboring the plasmid through hydrolysis of ampicillin.

Several possibilities can be considered to explain these observations in AM activity of STI.

First, it may interfere with plasmid transfer process to new (daughter) cells, thus making the new cells sensitive to ampicillin. Since, plasmids are also copied during cell division such that each daughter cell receive a copy of each plasmid, transfer of plasmid does not occur through conjugation with pili, syringe like cell surface structure, which should be accessible to STI. So this possibility can be eliminated.

Second, STI may inhibit lactamase enzyme, making the cells sensitive to ampicillin. Since, most β-lactamase enzymes contain a serine in their active site similar to serine protease (but lacks the aspartate and histidine of the catalytic triad)^40^, it is possible that STI to some extent can inhibit β-lactamase enzyme in a manner it inhibits trypsin or chymotrypsin. Unable to destroy ampicillin, the cells will die. Further investigations are necessary to rule out this possibility.

Third, STI may interfere with β-lactamase synthesis, making the bacterial cells sensitive to ampicillin. This is possible if STI nicks the plasmid at a regulatory site or at open reading frame of β-lactamase gene as we observed in *in vitro* experiments of this study, leading to reduced replication or transcription of the plasmid (Fig. 6B,C,E). This will make the cells vulnerable even with the *Amp*^*r*^ plasmid. In order to inactivate plasmid, STI or its fragments, if digested by bacterial enzyme, has to enter *E. coli* cells crossing cell wall, and outer and inner membranes.

The thin cell wall in these cells is not a barrier to solutes, the openings in the mesh are large and almost all types of molecules can pass through^38^. In order to inhibit lactamase enzyme in possibility 2, STI must cross outer membrane and reach periplasm. Now, can STI cross the inner membrane? Deletion of the two Ile resides in P18–26 (IIRYFYNAK) of aprotinin completely destroyed its AM activity, suggesting the hydrophobicity was required, which was further demonstrated when a hydrophobic pentapetide (FFVAP) was linked to its C-terminus, the AM potency was increased remarkably^38^. These suggest that insertion into membrane or crossing the membrane into cytosol seems an important factor for AM activity. It is possible the significant variation in AM activity among various inhibitors (Table 1) may reflect how efficiently they get into bacterial cell. Electron microscopy of ORB-treated cells discovered formation of several large mesosomes in the cytosol^25^. It also identified an unknown condensed structure most likely made of DNA in the cytosol. Inspection of the TI domain in STI showed nearly 60% (18 residues) are hydrophobic. The observation in this study that STI nick plasmid DNA may provide support for this 3^rd^ possibility.

As a PI, aprotinin’s physiological functions include protective inhibition of trypsin when small amounts are produced by cleavage of the trypsinogen precursor during storage in the pancreas. However, even after so many years, its physiological function for AM activity was not fully known^38^, yet it is found in the blood stream with another inhibitor, α anti-trypsin. Likewise, protease inhibitors in plants, especially in seeds, serve as an anti-nutritive factor on whomever feed on them, but little is known about biological roles of their AM activity in plants. Most microbes secrete proteases that may play role in disease progression, thus combating these proteases by PIs manifested as AM activity seems logical. However, the fact that not all PIs possess AM activity argues against this rationale and suggests additional mechanisms exist.

In a recent study, aprotinin was shown to target magnesium transporter Alr1p in Candida albicans to lower cellular Mg^2+^ levels resulting in fewer cells in S-phase of the cell cycle and a corresponding increase of cells in G(0)/G(1) and G(2) phases^15^. The novel finding in this study that STI and bovine aprotinin nick plasmid and significantly lower its infectivity adds yet another mechanism. The finding that STI is more effective against plasmid harboring bacteria and prediction of an unknown condensed substance in the cytoplasm of TI treated bacteria as DNA was the 1^st^ proposed direct effect of PIs on DNA and lends a support to our finding.

## Materials and Methods

Both BioUltra Soybean trypsin Inhibitor (T2327) and bovine pancreatic Aprotinin (A1153) were obtained from Sigma Aldrich (St. Louis, Missouri, USA) as lyophilized powders with ≥98% purity. They were dissolved in sterile water and stored in −20 °C in aliquots to avoid repeated freeze-thaw. The products remained active in frozen aliquots at −20 °C^28^.

The DNA used in all experiments was pGL3 DNA (2867 bp) or its modified version was was amplified in *Escherichia coli* XL-1 blue cells transformed by the plasmid in 5 ml culture in LB broth supplemented with antibiotic ampicillin. Plasmid was purified using GenElute plasmid miniprep kit (Sigma) following manufacture’s instruction and analyzed by gel electrophoresis on 1% agarose gels in 1x TAE (40 mM Tris, 20 mM Acetate and 1 mM EDTA, pH around 8.3).

### DNA nicking reaction

pGL3 plasmid in Buffer A (25 mM Tris-acetate, pH 7.5, 100 mM potassium acetate, 10 mM magnesium acetate) was incubated with treated or untreated STI (1 to 3 μg) at 37 °C for 0–2 hr. Some reactions were incubated in the presence of NaCl (0–0.5 M). In others, EDTA (0–15 mM) was included. Following incubation, the reaction mixtures were mixed with 5 × loading buffer (100 mM Tris-HCl, pH 8.0, 5 mM EDTA, 30% glycerol, 0.25% BPB) before being analyzed by electrophoresis in 2% agarose gel. The gel was run in 1x TAE at 60 volt for 45 min.

### Various treatments of STI

STI (15 μl, 3 μg/ul) in separate tubes was boiled for 5, 10, 20, 30 and 60 min at 90 °C and then cooled on ice for 5 min before used.

STI (15 μl, 3 ug/ul) in separate tubes was treated with DTT (0 or 5 mM), 2-mercaptoethanol (2.5 or 5%) or EDTA (0-10 mM) for 1 hr at room temperature.

STI (50 μl, 3 μg/μl) dissolved in 20 mM acetate buffer, pH 2.0 was incubated at 4 °C for 12, 24 and 48 hr.

STI ((20 μl, 3 μg/μl) of STI was digested with 5 μg of trypsin at 37 °C or 5 μg of proteinase K at 60 °C for 2 hours. BSA was used as a control for digestion with these enzymes. A portion of trypsin treated STI was incubated with 10 mM EDTA for 10 min prior to use in plasmid nicking assay.

All treated samples were stored at −20 °C until further analysis. Solutions are stable in frozen aliquots at −20 °C.

RNA (1 μg) isolated form HepG2 in RIP buffer (167 mM KCl, 100 mM MgCl_2_, 100 mM Tris-HCl, pH 7.2) was incubated with 3 μg of RNase, STI or none. The mixture was resolved on a 2% agarose gel stained with ethidium bromide.

The treated and untreated DNAs were prepared using MiniElute PCR purification Kit (Qiagen), followed by gel quantification. Then equal amount of DNA was used for transfection of HEK293T cells.

### FPLC

Fast protein liquid chromatography was carried out on Mono Q column in a BioRad instrument with a UV detector set at 220 nm in 50 mM Tris-HCl, pH 8.0 buffer with a linear gradient of 0.5 M NaCl in the same buffer. Fractions (0.5 ml) collected were concentrated using Speedvac at 35 °C under vacuum to 50 μl.

### Trypsin inhibition assay

To assay, 10 μl of trypsin (10 μg) was added with 1 μl (3 ug) treated or untreated STI. Then, 10 μl of the substrate, *N*α-Benzoyl-DL-arginine 4-nitroanilide hydrochloride in PBS, pH 7.4 was added and incubated at 37 °C for 10 min, and absorbance was measured at 415 nm in a BioRad plate reader.

### Transformation

Following incubation without or with inhibitors, the DNA were purified and 50 ng of the purified DNA was used in transformation of XL-1 blue competent cells using heat shock at 42 °C. Following recovery, 1/10th of the transformation reaction was used for plating in LB ampicillin plate.

### Cell culture and transfection

Human embryonic kidney 293T (HEK293T) cells were cultured in Dulbecco’s modified Eagle’s medium (DMEM) with 4.5 g/liter glucose containing 10% (v/v) heat-inactivated fetal bovine serum (FBS) and antibiotic (100 IU/ml penicillin, 100 μg/ml streptomycin and were grown at 37 °C supplied with 5% CO_2_. Cells were transfected with 200 ng of the purified DNA using the calcium phosphate method.

### Antimicrobial activity

An overnight culture of *E. coli* was diluted 7% in fresh LB media and aliquoted 100 ul in sterile 96-well tissue culture plates. Cells harboring a plasmid was grown in LB with ampicillin (50 μg/ml). Various concentration of STI in 10 ul was added to each well. The Plate was covered with a sterile lid and incubated at 37 °C for 8 h with shaking at 125 rpm. Microbial growth was assessed by measuring increase in absorbance at 570 nm. Assays were repeated three times.

### Digital image and processing

The images were captured by Cannon Powershot G10 under UV light for DNA and white light for protein as jpg file, and cropped with Microsoft Photo Gallery. Samples in each gel were prepared in parallel. All gels were processed using Microsoft Word “Corrections” for brightness and contrast applied equally across the entire image including controls. The full length gels are presented in Supplementary Figures with corresponding numbers as in the main article.

### Quantification bands

NIH ImageJ software was used to quantify the slower moving band. The intensity for control was set to 1.0.

## Supplementary information

Full Length Gel Figures

## Data Availability

No datasets were generated or analyzed during the current study.
