# Supplementary material for: Plasmid DNA nicking- a Novel Activity of Soybean Trypsin Inhibitor and Bovine Aprotinin
Source: Sci Rep. 2019 Aug 12;9:11596. doi: 10.1038/s41598-019-48068-6 (PMC6690959; doi:10.1038/s41598-019-48068-6)

Supplementary Information File

Plasmid DNA nicking- a Novel Activity of Soybean Trypsin Inhibitor and Bovine Aprotinin

M. Rafiq Islam*, Kelvin Ihenacho Ihenacho, Jae Whan Park, I. Sakif Islam

Figure 1

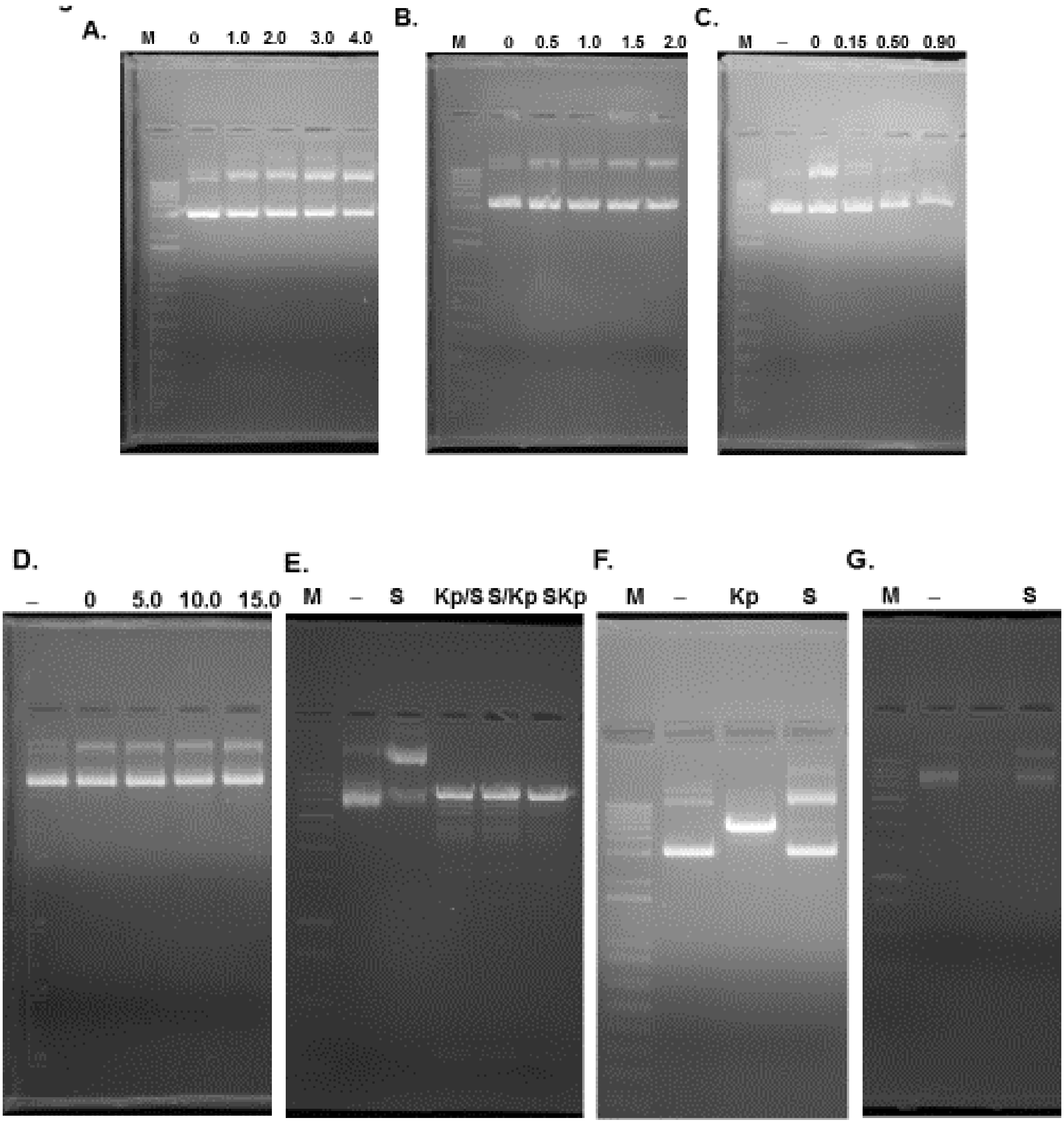

A.
M 0 1.0 2.0 3.0 4.0
B.
M 0 0.5 1.0 1.5 2.0
C.
M – 0 0.15 0.50 0.90
D.
– 0 5.0 10.0 15.0
E.
M – S Kp/S S/Kp SKp
F.
M – Kp S
G.
M – S

Figure 2

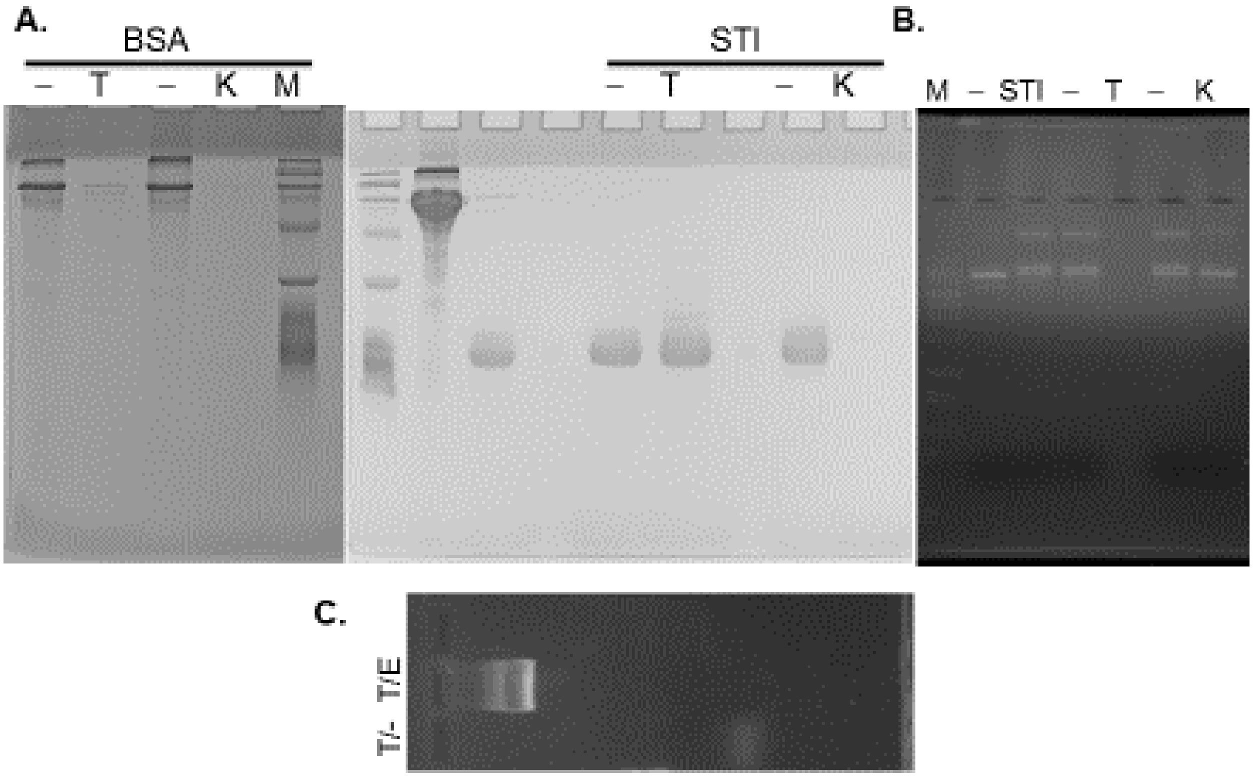

Figure 3

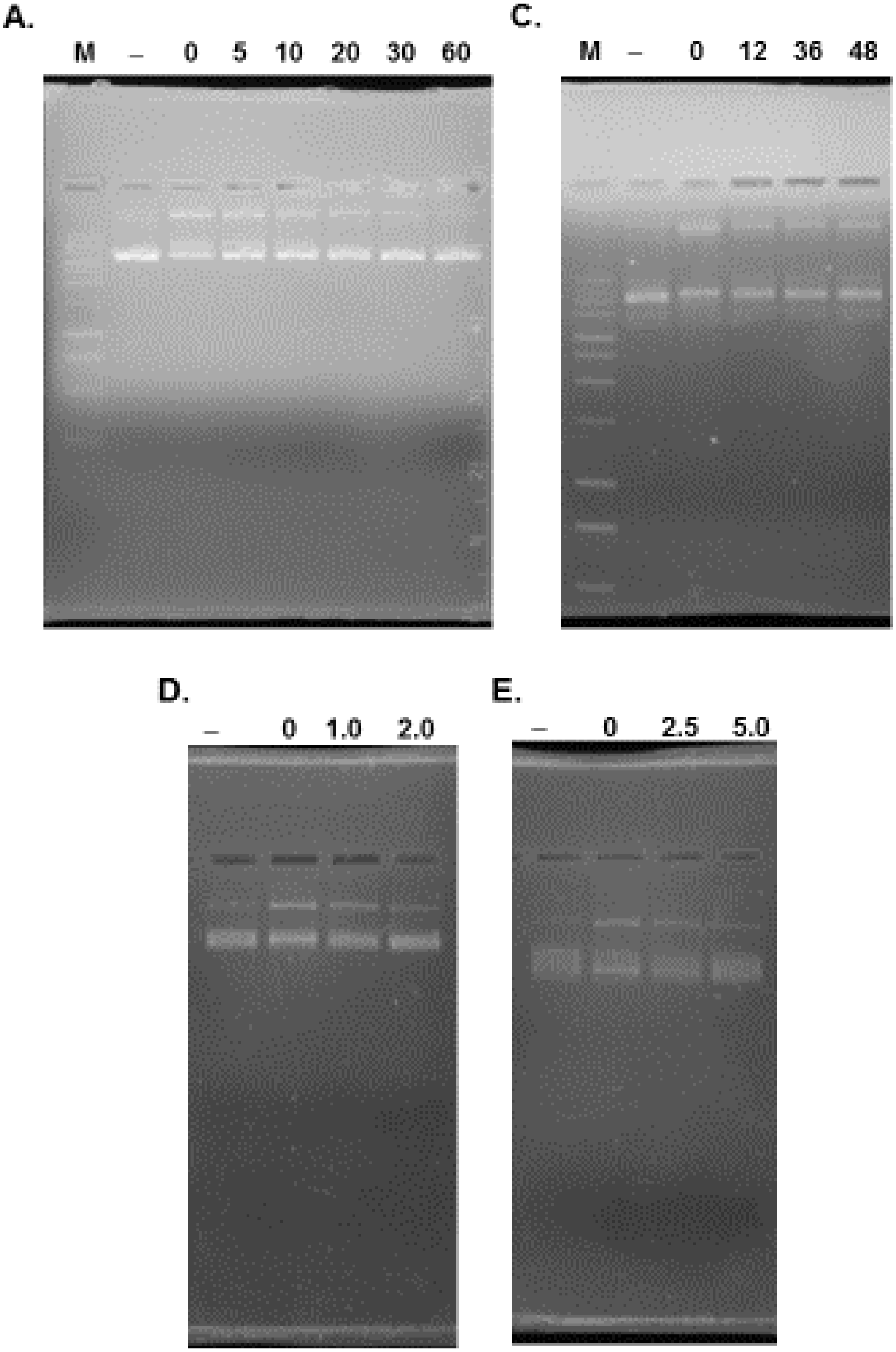

Figure 4

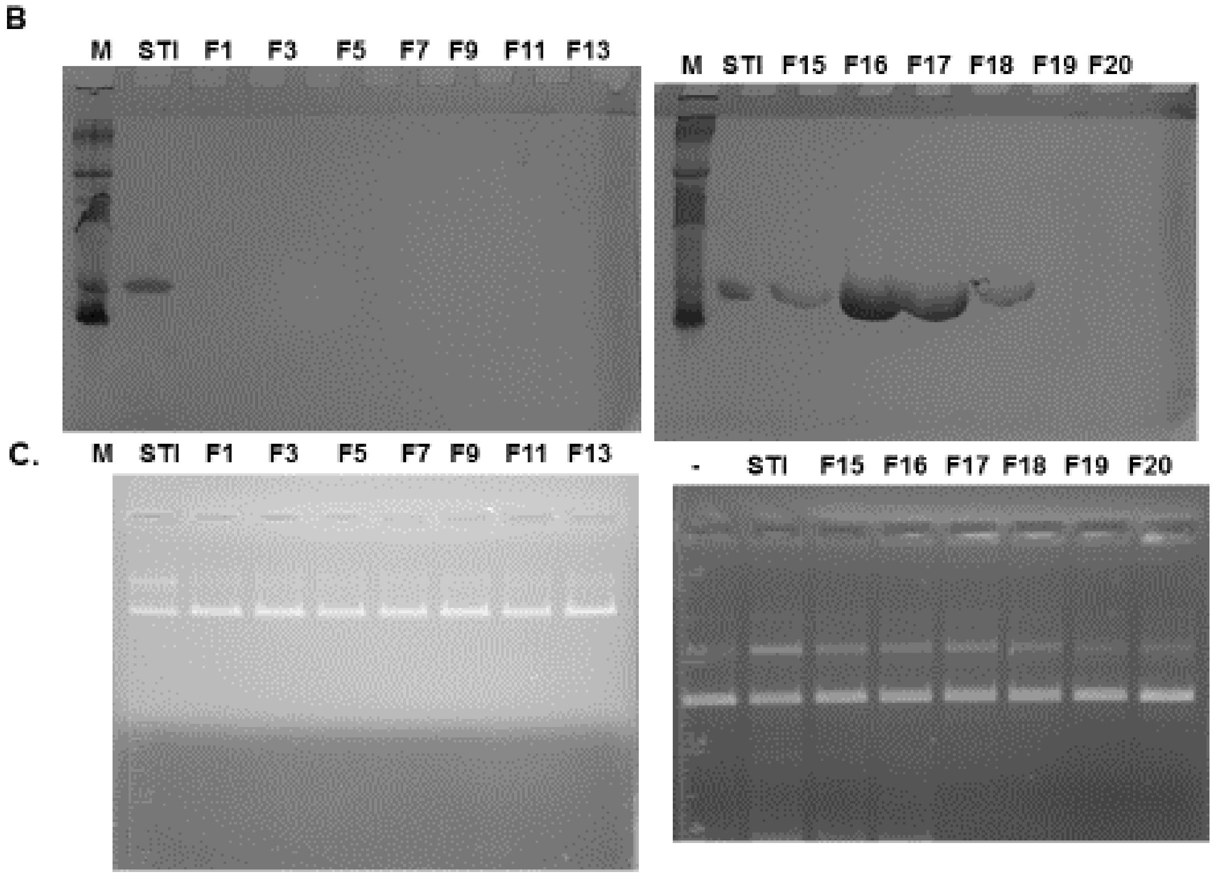
B
M STI F1 F3 F5 F7 F9 F11 F13
M STI F15 F16 F17 F18 F19 F20
C.
M STI F1 F3 F5 F7 F9 F11 F13
- STI F15 F16 F17 F18 F19 F20

Figure 5

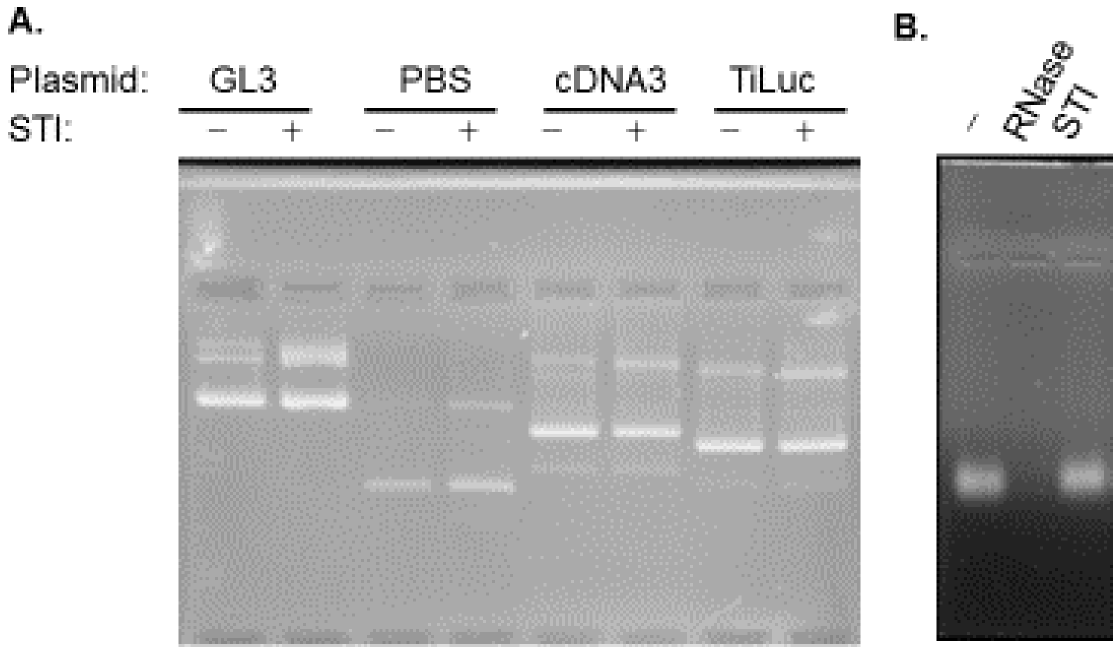

Figure 6

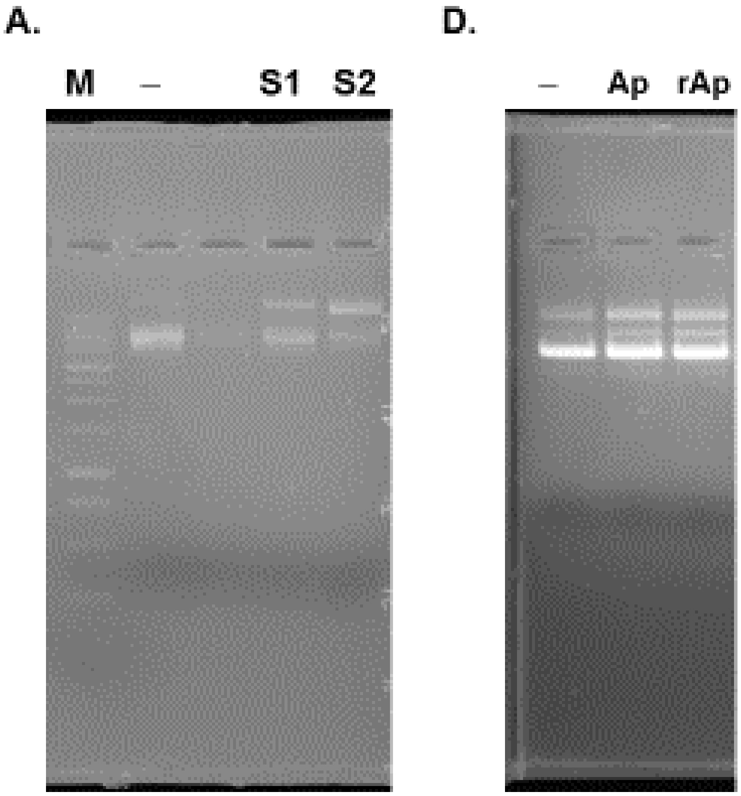

Supplement: Supplementary file 1 — Full Length Gel Figures [file 41598_2019_48068_MOESM1_ESM.pdf]
